# Design of a Mobile Application and Evaluation of Its Effects on Psychological Parameters of Covid-19 Inpatients: A Protocol for a Randomized Controlled Trial

**DOI:** 10.3389/fpsyt.2021.612384

**Published:** 2021-05-24

**Authors:** Shokoufeh Aalaei, Farnaz Khoshrounejad, Lahya Afshari Saleh, Mahnaz Amini

**Affiliations:** ^1^Department of Medical Informatics, Faculty of Medicine, Mashhad University of Medical Sciences, Mashhad, Iran; ^2^Department of Occupational Medicine, Faculty of Medicine, Mashhad University of Medical Sciences, Mashhad, Iran; ^3^Lung Diseases Research Center, Faculty of Medicine, Mashhad University of Medical Sciences, Mashhad, Iran

**Keywords:** mobile health (mHealth), telemedicine, patient education and consulting, psychology, COVID-19, telemental health, telehealth, information technology

## Abstract

**Background:** Panic of the disease and the associated concerns can lower the quality of life and physical performance. As long as the COVID-19 pandemic is ever on the rise, the psychological pandemic of the disease is on the rise, too. The high prevalence of COVID-19 has further increased physicians' work pressure. Patients' needs are not met adequately by physicians. It seems essential to use aids to monitor patients' needs and serve them properly. Thus, in the present research, suggestions are made on how to evaluate patients' physical and psychological conditions during the treatment via a mobile application.

**Methods and Analysis:** The present research is a randomized, two parallel-group, controlled trial. One-hundred-twelve inpatients diagnosed with the coronavirus will be assigned randomly to the control and intervention groups. In the intervention group, a mobile application will be provided to educate patients, establish two-way interactions between patients and care providers and record patients' symptoms. Those in the control group will receive the usual care. The primary outcome is the change to the depression anxiety stress scales-21 (DASS-21) score from the baseline to 2 weeks after discharge from hospital. It will be measured at the baseline, at the time of discharge, and two weeks later.

**Ethics and Dissemination:** The Ethics committee of Mashhad University of Medical Sciences' approval date was 2020-04-19 with IR.MUMS.REC.1399.118 reference code. Thus far, participants' recruitment has not been completed and is scheduled to end in March 2021. The results will be disseminated in a peer-reviewed journal.

**Trial Registration:** IRCT20170922036314N4 (https://www.irct.ir/trial/47383).

## Introduction

Panic of the unknown can reduce perceived security in humans, and it has always been stressful. The prevalence of COVID-19 can be stressful too. The symptoms of the disease can range from mild to severe. The symptoms include fever, coughs, and shortness of breath (1). Anxiety is a common symptom in patients afflicted with a chronic respiratory disease and can lower the quality of life to a great extent. Clinical anxiety plagues about two-third of respiratory patients and also reduces the quality of life and physical performance. Anxiety induced by Covid-19 prevails and it seems to originate from the unknown nature of the disease and the ambiguity involved. Panic of the disease and the associated concerns might be so intense that it can provoke patients, adults and children. Often, people at a higher risk of the disease (those with diabetes, a transplant, hypertension, respiratory problems, etc.) react more severely to the disease (2).

Because COVID-19 pandemic is ever increasing, the psychological pandemic of the disease is also on the rise. New psychological problems have emerged due to the unreliability and instability of the coronavirus, bad news on the rapid transmission of the disease among people (3, 4), the unknown origin of the virus (5, 6), its high reproductivity (6) and the unpredictability of conditions. Besides the physical suffering, the increased mental pressure involved in confirmed cases of the disease or suspicious circumstances can exacerbate the condition (7, 8). Anxiety and panic can worsen the shortness of breath and reduce patients' adherence to physicians' advice and can even worry the medical staff. If so, patients may experience loneliness, denial, anxiety, depression, sleeplessness, and disappointment. This can reduce the rate of adherence to medical treatment. Some cases may even increase the chances of aggression and suicide (9). Besides inpatients, those discharged also experience different degrees of stress even after the end of the event.

On the other hand, at present, the high prevalence of COVID-19 has further increased physicians' work pressure. Patients' needs are not met adequately by physicians. It seems essential to use aids to monitor patients' conditions and serve them properly. As previously mentioned, COVID-19 patients are affected both physically and psychologically. They need support from both sides.

The current pandemic makes it essential to explore the different aspects of psychiatry, including social-psychological and medical interventions, to find effective evidence-based treatments. One way to reduce patients' stress is to run medical programs and report the health state to them, which can be provided in multiple ways (10).

The past decades have witnessed advanced digital technologies and, thus, the relevant revolutions are pretty promising. New technologies can cost-effectively improve health service provision. Today, many people can access such technologies at a low cost, especially technologies based on smartphones and mobile applications, which have attracted the global population. Thus, it seems that mobile-based interventions play a key role in providing medical services during the pandemic.

Mobile health solutions can potentially provide valuable services during the COVID-19 pandemic. These services can be in the form of education, resource allocation, surveillance, screening, treatment, diagnosis, prevention, management, and control (11).

Applications for contact tracing, symptom monitoring and information provision are among the critical applications developed to manage COVID-19 pandemic (12). Though many applications have been developed for COVID-19, many do not have the right quality (11).

A significant body of research has dealt with the effectiveness of self-care applications and software from physical and psychological aspects in different patients (13–16).

A body of research explored the role of mobile applications in mental health (17, 18). The findings of these studies along with the results of a review in 2019 (19) show that applications play a key role in reducing the symptoms of depression. Besides the patients, physicians' attitude toward the use of mobile-based interventions is positive and promising. Physicians' and patients' acceptance of mobile applications in this field contributes to the use of mobile-based devices for self-management in mental diseases. Due to the notable limitations of the pandemic (e.g., the quarantine, less social contact, and consequently less face-to-face referral to care-providing centers), attention has been drawn to telehealth approaches to facilitate the mental health-related services (20–23). Due to the effectiveness of mobile-based interventions in mental health domain, during the pandemic, mobile-based interventions can be used as non-drug interventions with almost no adverse effect on the COVID-19 patients. These can help reduce depression and anxiety (24).

Thus, in the present research the aim is to evaluate patients' physical and psychological conditions during the treatment (both during hospitalization and recovery) via a mobile application. To this aim, the data stored in the application which represent the patients' condition during the disease will be analyzed by a team of experts consisting of physicians and psychologists. It is expected that removing patients' ambiguities by teaching medical and psychological content, monitoring patients during hospitalization and recovery, and sending them feedback by the physician help reduce mental pressures and improve the medical treatment.

## Methods

### Study Design and Setting

**Figure 1:**
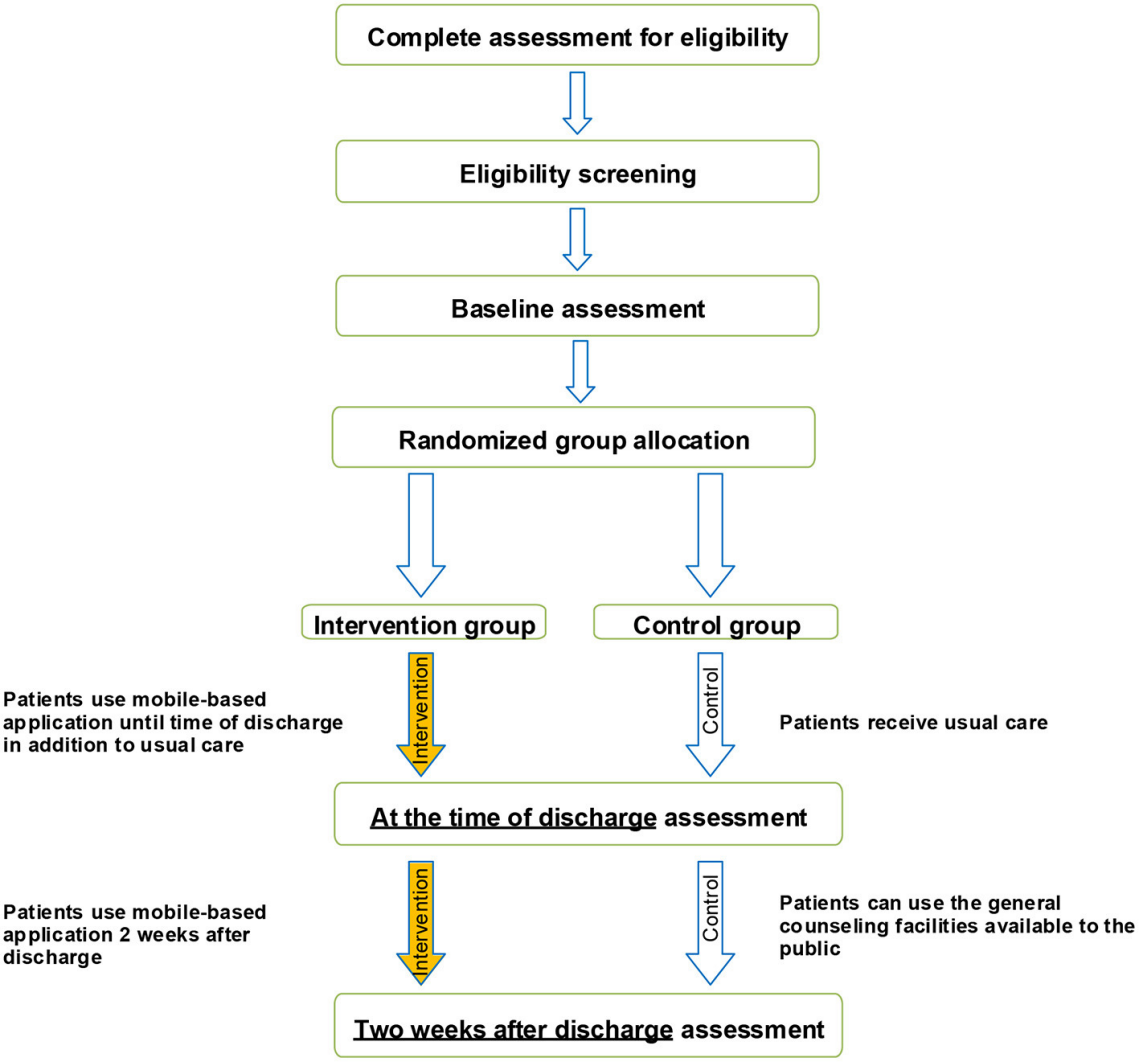
Schematic of the study procedure.

A randomized, two parallel-group, controlled trial is designed following the Consolidated Standards of Reporting Trials guidelines (25) (Figure 1) to investigate whether the mobile application designed will make any difference in the emotional states of depression, anxiety, and stress in target patients and whether it will produce any better outcome in COVID-19 patients.

The study will be conducted in Imam Reza Hospital in Mashhad, Khorasan Razavi Province, Northeastern Iran. The center is affiliated with Mashhad University of Medical Sciences (MUMS). This hospital has been a referral center for COVID-19 patients since the start of the pandemic in Iran.

### Participants

The target population consists of patients hospitalized with the coronavirus. The eligible patients, based on the inclusion criteria, will be informed about the purpose of study. Then an assistant researcher will collaborate to obtain the written informed consent. It will be ensured that participants can withdraw from the study any time they wish with no effect on their subsequent care. The informed consent has already been evaluated by the Ethics Committee of MUMS (Ethical code: IR.MUMS.REC.1399.118).

#### Inclusion Criteria

- baseline literacy
- using a smartphone
- ability to work with a smartphone.

#### Exclusion Criteria

- unwillingness to continue with research
- transference to the wards where it is not possible to use a mobile phone, such as the intensive care unit
- deterioration of the patient's physical condition.

### Objectives

The primary purpose of the present trial is to investigate the effectiveness of a mobile application in improving hospitalized Corona patients' psychological parameters such as depression, anxiety, and stress. The trial will also assess any change to respiratory/non-respiratory symptoms in the control and intervention groups.

### Control Group

Participants in the usual care group will receive standard care as provided by their physicians at the time of hospitalization. After discharge, they will be recommended to use the general counseling facilities available to the public as soon as required.

### Intervention Group

All patients participating in the intervention group will have to install a user-friendly mobile-based psychological counseling and health status monitoring application named “SarveDigital” on their mobile phones. They will also receive usual care the same as the control group.

Participants in the intervention group will be provided with oral and printed instructions for accessing and logging into the application. To access the application, it is necessary to fill in a personal form. Once this is adequately completed, the app services can be accessed.

Participants will be advised to have full access to the program until 2 weeks after discharge, and will be encouraged to use the program regularly. They will be recommended to use the educational content of the application and ask their questions from a physician via the application.

### Intervention

#### Need Analysis

In designing the application for COVID-19 patients, the first step was to extensively review the related literature on the pre-existing applications in terms of effectiveness, appropriateness to the target group, specific features, and probable discussions in design. Then, in several meetings with internal specialists, psychologists, sports consultants, and medical informaticians (no. of experts = 5), the scientific aspects of the topic, patients' needs during hospitalization and after discharge and the necessity of offering complementary solutions were analyzed. Consequently, according to the present findings, experts' comments and patients' cultural and local tendencies, the main features of the application were specified for the design.

#### Design Process

In order to design the technical features of the application and apply the maximum capabilities of the mobile application production domain, a team of experts was consulted. The original idea of the application was discussed in meetings shared between the clinical and technical teams. The ambiguities were solved and the technical aspects were discussed.

Eventually, considering the fact that the main goal of developing the application is establishing interactive communication between patients and healthcare providers, a mobile application was designed to support patients physically and psychologically.

During the design procedure, a formative evaluation was used and the feedback received from the experts and sample patients helped to remove the existing defects. The major changes to the application were: change in content, content categorization, larger font size, and change from a native app approach (Android-based application) to a web-based approach (web-based application) due to its compatibility with all smartphones regardless of the operating system. It will be possible to use the application for more patients.

#### Application Features

The mobile application has been designed with a simple, intuitive user interface.

**Figure 2:**
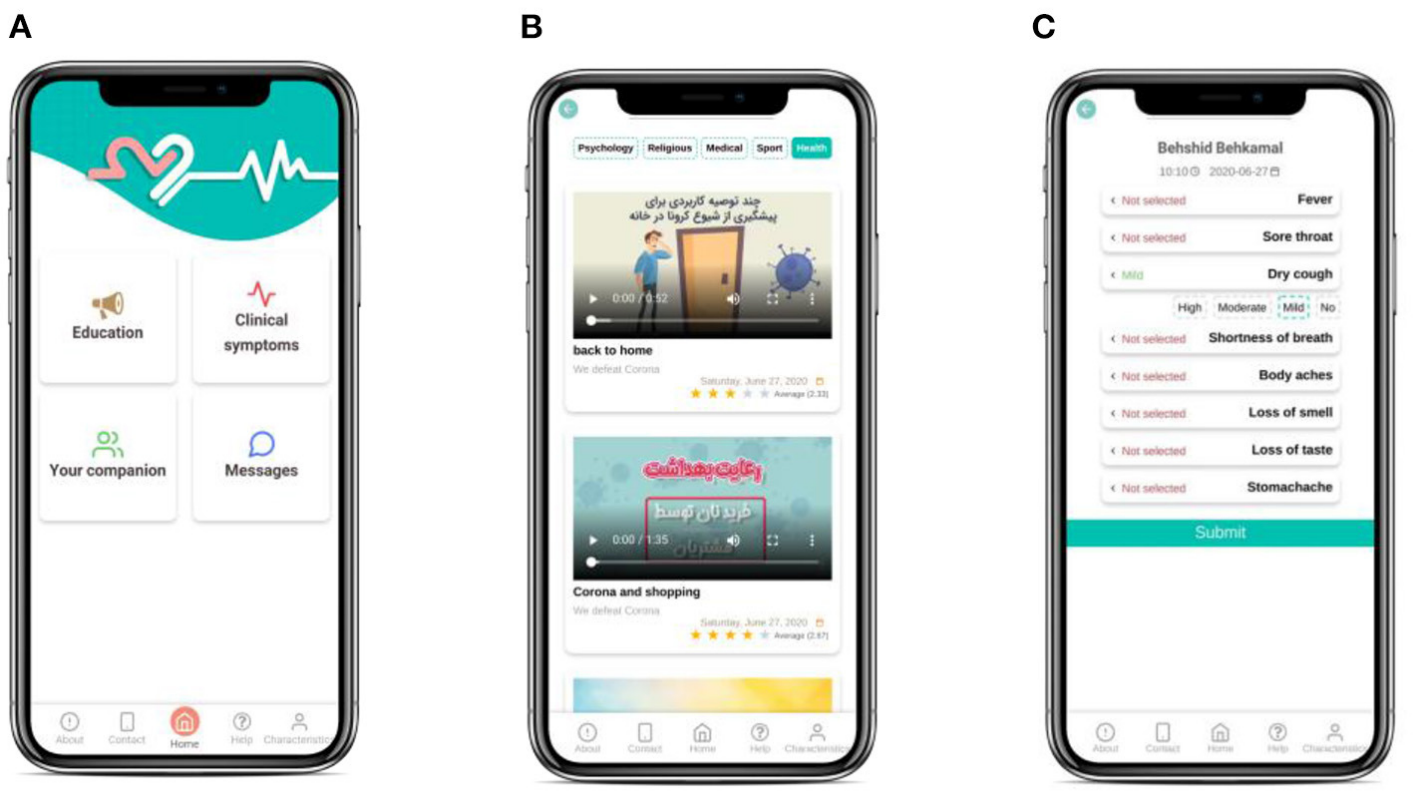
Screenshot of **(A)** home page view, **(B)** education, and **(C)** symptoms self-report.

The application consists of 4 main parts (Figure 2):

- **Education:** This part consists of a broad range of educational content during hospitalization and after discharge including psychological, general exercise, medical and health videos and texts about COVID-19 patients. The educational material required for COVID-19 patients will be provided using valid educational resources and experts' consensus.
  To improve the content in later versions of the software, at the end of each educational medium, the patient will be asked to rate the content and help us increase the quality of services.
- **Contacting Care Providers:** This part serves the following functions:
  1. Support by other medical staff besides the attending physicians such as psychologists who cannot be physically present in the clinic for safety matters.
  2. Patient-physician communication via software in hours later than daily visits. Besides filling out an improvement checklist, the patient can write a text about personal queries and concerns.
  3. Physician's (or any other therapist's) feedback for patient. Upon seeing the patient's text, the therapist can send a text-based or audio message as the response (to relieve the patient).
    Contacting care providers will be disabled at the end of the study (2 weeks after discharge).
- **Symptoms Self-report:** SarveDigital will help COVID-19 patients record their symptoms and, thus, their physician will be able to monitor and control their condition from a distance. Patients will be asked to enter their health symptoms such as fever, sore throat, dry cough, difficulty breathing or the shortness of breath, and loss of taste or smell at least three times a week into the application. Each symptom will be rated on a 4-point scale (e.g., “no fever,” “mild fever,” “moderate fever,” and “high fever”).
  These data will automatically be sent to the physicians' portal so that the physician will be aware of the patient's health status. It should be noted that the attending physician and the psychologist assigned will be able to access physicians' portal via their mobile phone with a username and password. This will ensure the privacy and security of the patient's information and the relevant classified information (26).
- **Care Providers' Biography:** This part introduces the key information about the attending physician and psychologist who will communicate with the patient. The aim is to increase the trust the patient places in them to easily share concerns, queries and else.

### Outcome Measures

#### Primary Outcomes

The primary outcome will be a change to the patient's depression anxiety stress scales-21 (DASS-21) score from baseline to 2 weeks after discharge from hospital. It will be measured at the baseline, upon discharge and 2 weeks afterward. It should be noted that baseline measurements will be collected as soon as the patient is admitted to the hospital. This will ensure that almost all patients are included under the same conditions.

#### Secondary Outcomes

- Changes to the respiratory symptoms including the respiratory rate will be determined through physician assessment; oxygen saturation will be measured through pulse oximetry; lung involvement will be checked by imaging at the time of discharge.
- Changes to the non-respiratory symptoms including multi-organ failure will be determined through kidney and liver function tests; fever will be measured with a thermometer; secondary infection will be measured by physician assessment at the time of discharge.

### Data Collection Instruments

Psychological assessment will be conducted using the DASS-21 questionnaire, as the primary outcome. This questionnaire (21 items) is primarily designed to test emotional states such as depression, anxiety, and stress with three respective subscales each with 7 items. The instrument is known for appropriate psychometric properties and is widely employed. A validated version of the questionnaire in Persian language (27) will be used in the present study with three subscales as the main indicators of the negative emotional and psychological responses. A 4-point Likert scale will be used to test the emotional state of each subscale in the questionnaire. The scale ranges from 0 (does not apply to me at all) to 3 (applied to me very much, or most of the time). As for the total score of each subscale, it ranges from 0 to 21, and a higher score represents a higher level of depression, anxiety, and stress. According to Asghari et al. (27), Cronbach's Alpha test of internal consistency was estimated at 0.94 for the whole scale and 0.85, 0.85, and 0.87, respectively, for depression, anxiety, and stress subscales. Moreover, the intra-class correlation with an absolute agreement between Time 1 and Time 2 assessment occasions for depression, anxiety and stress scales were found to be 0.77 (95% CI: 0.56–0.88), 0.89 (95% CI: 0.81–0.94), and 0.85 (95% CI: 051–0.94), respectively. Intra-class correlation values above 0.74 represent a good reliability (28).

The secondary outcomes (i.e., respiratory/non-respiratory symptoms) will be determined by physician assessment, pulse oximetry, radiology images, kidney and liver function tests, and a thermometer.

- In the intervention group, the degree to which the software is used (e.g., seeing the educational content, completing symptoms) and the quantity of questions from care providers will be extracted from the recorded logs of every user and will be reported accordingly.
- At the end of the research, the survey on the quality of application will be submitted to patients in the intervention group. The information will help to improve the later versions of the application. The questionnaire, derived from the related literature in telemedicine (29, 30), will enquire about users' comments on content domains, presentation design, ease of use, usability and satisfaction. Translation, modification and usage of the questionnaires were based on the opinion of three experts in the field of medical informatics familiar with telemedicine research. After an initial review of the content (of the questionnaires) by the experts, a limited sample of the patients (*n* = 5) also evaluated the instrument to further revise it.

All study content, screening and questionnaire data will be collected through online survey software (Google Form).

### Sample Size

The similar research was used to estimate the sample size. The most similar study to the present work was conducted by Proudfoot et al. (31), who explored the effect of a mobile application on stress, anxiety and depression. In the above-mentioned research, after the intervention, the effect size between the control and intervention group (the one provided with the application) was reported to be 0.41. The sample size is estimated 190 in total (95 for the intervention group and 95 for the control group) which is calculated in G^*^Power software with an effect size of 0.41, error probability of 0.05, power of 0.8, and allocation ratio of 1:1. Regarding the 10 % attrition rate within the study, the goal is to include 105 patients in each group (210 participants overall).

### Randomization

www.randomization.com will be used to create a randomization sequence. The sequence of the generated random number will be inserted into envelopes sealed by an independent researcher (SA), not participating in the main data collection phase or in the intervention. These envelopes will be opened only after the participants sign the informed consent and complete all baseline assessments (FK). Then, the eligible participants will be randomly divided into two groups, a usual care group and an intervention group, which uses the application.

### Blinding

The nature of the intervention is in a way that does not allow for patient blinding. Similarly, the outcome assessor is not blinded either. That is because s/he is constantly in touch with patients in the intervention group to answer the application questions. The data manager (who generates the randomization sequence, prepares envelopes and maintains a list of the participants who already enrolled) and the data analyzer will be completely blinded to the control and intervention group assignment and specifications.

### Data Management

A management committee (MA, SA, and LAS) is formed to monitor data quality and to approve any decision in the present trial. Data entry and coding will be conducted by people other than the main research team and will be checked by the management committee through range-checks for data values.

### Statistical Analysis

The normal quantitative variables (checked via the Kolmogorov–Smirnov normality test) will be described using mean and standard deviation. The rest of data will be described using median and interquartile range. To compare the two research groups in terms of the mean difference of quantitative variables, if the normality assumption is met, an independent-sample *T*-test will be run. Otherwise, Mann–Whitney *U*-test will be employed. To test the homogeneity of qualitative variables in the two groups, the Chi-squared test and Fisher's exact test will be run at the *p*-value of 0.05. Repeated-measures or equivalent non-parametric tests will be employed for the continuous outcomes measured repeatedly. All these tests will be two-tailed at a 5 % significance level. Statistical analyses will be done in SPSS22. The analysis will be conducted on an intention to treat. No interim analyses are planned.

## Results

So far, the design procedure has been fulfilled. The recruitment of participants has not been completed and is scheduled not later than March 2021.

## Discussion

The present research, a randomized clinical trial in type, aims to evaluate a mobile application designed to monitor COVID-19 patients and provide them with clinical and psychological feedback compared to usual care. It seems that such an intervention can provide proper support for the peak time of the disease occurrence. The prospective findings will provide evidence for the effect of mobile health on COVID-19 patients. This research, which will be conducted by an interdisciplinary team, makes it possible to evaluate the effect of this intervention precisely.

Mobile-based interventions can be effective in all stages of prevention, treatment, and follow-up. Instances are checking symptoms, educating patients, communicating with patients and adhering to treatment. Different studies have dealt with the effect of mobile applications on stress and anxiety management and managed to control them (14–16). Thus, it seems that providing psychological support for COVID-19 patients can reduce mental pressure, signs of depression and anxiety (32).

Providing mental healthcare services is not only essential for patients with COVID-19 but also for all individuals who are at a higher risk of the disease. Preliminary reports show that, during the pandemic, people are actively searching for online services to meet their mental health needs. It proves public interest in and acceptance of the medium (33). China, as the first country faced with the coronavirus, has been actively involved in providing different telemental health services during the pandemic. These services have been provided by academic and governmental organizations and include online counseling, supervision, training, and psychoeducation. Research findings proved the effectiveness of online telemental health services and social networks in improving patients' tolerance and stress (34).

Among the benefits of telehealth services are enabling individuals to welcome treatments, cooperate in diagnostic and therapeutic measures, and giving them personal feedback, and supporting or motivating them (35).

The three factors of physician, patient and disease play a key role in the therapeutic process of a disease. If the first two are united, they can pass through the third (i.e., the disease). To unite the physician and patient, it is essential to create connections and interactions between them, possibly facilitated by mobile-assisted technologies (36). We set this issue as a main goal in SarveDigital development. Thus, the instrument developed will enable patients to report their clinical and psychological problems to the medical staff at almost any time. Moreover, this two-way communication will help patients to be actively involved in the treatment procedure, as advised by the physician. There is no harm in using a mobile phone by COVID-19 patients and this is a right preserved for them.

The majority of patients are only examined once a day by the attending physician. Therefore, reporting the respiratory, physical, and mental conditions to the attending physician can create a sense of trust in the medical system and can reduce anxiety. It is in line with the results of a study by Noee et al. (37) which investigated the potential applications of mobile apps in the Iranian health system based on physician opinions. In the light of the present findings, to further develop mobile-assisted healthcare services in Iran, the priorities are: recording and tracking vital signs, supporting electronic decision-making in health-related issues, providing healthcare for remote areas, improving service quality, selecting the best health behavioral patterns, raising social awareness and improving health-related behavior, and increasing patients' knowledge of the disease (37).

It seems that, within the complications of COVID-19 pandemic, most of the above-mentioned priorities should be considered in the applications designed so that we can hope to manage the disease.

An investigation of the existing healthcare applications in Iran shows that the design and development of mobile health applications have grown at least quantitatively in recent years. Considering the fact that Iran is among the top-ranking Middle-eastern countries using mobile phones, it is not far from expectation to warmly embrace the health-related applications (36). A review of published literature on mobile health in Iran proves that the mobile health-related body of research is ever increasing in Iran (38). Although Iranian researchers joined this line of research later, the effectiveness of the interventions in this field in Iran further attests to the potential value of the interventions in disease management. COVID-19 pandemic drew further attention to the significance of using telemedicine tools. During the COVID-19 pandemic, Iranian people have been as psychologically pressed as all around the globe (39). More cases of mental health problems have been reported among COVID-19 patients than others (20) which shows the need for systematic and goal-oriented interventions for COVID-19 patients to support them both psychologically and physically.

If SarveDigital proves effective in telemonitoring COVID-19 patients, we can hope such instruments can reduce the medical staff's work pressure, especially those in the front line of battling the disease. It can also help to reduce COVID-19 patients' anxiety and stress.

## Strengths and Limitations of this Study

- The self-care mobile application in the present research was designed in a structured way with the help of a multi-disciplinary team.
- The application addresses specific, well-defined characteristics, including care-providers' education, contact, self-reported symptoms and care-providers' biography. These all can increase the chances of success.
- The design of this study (randomized controlled trial) tends to meet the highest level of evidence.
- We cannot blind patients to the nature of the intervention, and this is an alleged limitation in the present research.
- Some limitations of the present research are the low literacy of using mobile health in the target population and some patients' unwillingness to continue using the application.

## Ethics and Dissemination

The approval was obtained from the Ethics committee of Mashhad University of Medical Sciences on 2020-04-19 (# IR.MUMS.REC.1399.118). This trial was registered in Iran Trial Registrar under IRCT20170922036314N4 registration number and 22 June 2020 registration date. The results will be disseminated in a peer-reviewed journal.

The dataset supporting the present results can be made available by the corresponding author upon reasonable request. Personal information about the potential and enrolled participants will be saved on a secure file server research drive at MUMS to ensure confidentiality before, during and after the research.

## Data Availability Statement

The original contributions presented in the study are included in the article/supplementary material, further inquiries can be directed to the corresponding author/s.

## Ethics Statement

This study was approved by the Ethics Committee of Mashhad University of Medical Sciences and Medical School (Ethical code: IR.MUMS.REC.1399.118).

## Author Contributions

MA and LS conceived the study idea and design. SA and MA designed the plan of RCT implementation. FK conducts the RCT. SA and FK drafted the manuscript. All authors have been involved in critically revising the manuscript. All authors read and approved the final manuscript.

## Conflict of Interest

The authors declare that the research was conducted in the absence of any commercial or financial relationships that could be construed as a potential conflict of interest.
